# Nutrition and the Risk of Alzheimer's Disease

**DOI:** 10.1155/2013/524820

**Published:** 2013-06-20

**Authors:** Nan Hu, Jin-Tai Yu, Lin Tan, Ying-Li Wang, Lei Sun, Lan Tan

**Affiliations:** ^1^Department of Neurology, Qingdao Municipal Hospital, School of Medicine, Qingdao University, Number 5 Donghai Middle Road, Qingdao 266071, China; ^2^College of Medicine and Pharmaceutics, Ocean University of China, Qingdao 266003, China

## Abstract

Alzheimer's disease (AD) is a progressive neurodegenerative disorder that accounts for the major cause of dementia, and the increasing worldwide prevalence of AD is a major public health concern. Increasing epidemiological studies suggest that diet and nutrition might be important modifiable risk factors for AD. Dietary supplementation of antioxidants, B vitamins, polyphenols, and polyunsaturated fatty acids are beneficial to AD, and consumptions of fish, fruits, vegetables, coffee, and light-to-moderate alcohol reduce the risk of AD. However, many of the results from randomized controlled trials are contradictory to that of epidemiological studies. Dietary patterns summarizing an overall diet are gaining momentum in recent years. Adherence to a healthy diet, the Japanese diet, and the Mediterranean diet is associated with a lower risk of AD. This paper will focus on the evidence linking many nutrients, foods, and dietary patterns to AD.

## 1. Introduction

Alzheimer's disease (AD) is a progressive neurodegenerative disorder that accounts for the major cause of dementia in the world [1, 2]. The number of the disease is projected to reach 106.8 million worldwide by the year 2050; therefore, the disease is a growing public health concern with major socioeconomic burden [3].

 Much attention has been paid to disease-modifying factors and risk factors for AD [4]. Cognitive engagement and physical activities have been associated with decreased risk of AD, while diabetes, epsilon 4 allele of the apolipoprotein E gene (APOE *ε*4), smoking, and depression have been associated with increased risk of AD [5]. In recent years, there has been increasing evidence supporting the role of nutrition in AD [6–8]. A number of dietary factors such as antioxidants, vitamins, polyphenols, and fish have been reported to decrease the risk of AD, while saturated fatty acids, high-calorie intake, and excess alcohol consumption were identified as risk factors [9]. Dietary patterns, which better reflex the complexity of diet, have emerged in recent years to examine the relationship between diet and AD [10]. In this paper, we will investigate the evidence linking nutrients, foods, beverages, and dietary patterns to the risk of AD. 

## 2. AD Is Associated with Both Obesity and Malnutrition 

Obesity and overweight seem to be associated with AD [11–13]. However, the evidence relating obesity measured with body mass index (BMI) with AD is conflicting. Obesity (BMI > 30) in midlife has been found to increase the risk of AD, while late-life obesity was found to reduce the risk of AD [11]. Therefore, manipulation of adiposity may provide a means to prevent AD [14].

 Malnutrition and weight loss are frequent complications of AD, the mean prevalence of malnutrition in AD patients living at home is 5% as reported by Guigoz et al. [15]. Patients with AD had a worse nutritional status compared to that of controls [16], and a baseline lower nutritional status was reported to indicate the progression of AD [17]. In addition, weight loss was reported to predict rapid cognitive decline in AD patients [18], and treatment of weight loss and malnutrition may also be important in AD patients.

## 3. The Effects of Nutrients on the Risk of AD

Many nutrients, such as antioxidants, vitamins, fat, and carbohydrates, can affect the risk of AD. Although the mechanisms of these nutrients on AD are not clear, reducing the oxidative stress and amyloid beta-peptide (Aβ) accumulation is considered to play a role in the process of AD [19, 20].

### 3.1. Antioxidants 

The oxidative stress, the undue oxidation of biomolecules leading to cellular damage, promotes many studies of antioxidants in the prevention of AD [21].

#### 3.1.1. Vitamin A and β-Carotene

Vitamin A and β-carotene could be key molecules for the prevention and therapy of AD, due to their ability to inhibit the formation of both Aβ oligomers and fibrils [20]. It has been shown in vitro that vitamin A and β-carotene have antioligomerization effects on Aβ [22]. Low serum and plasma concentrations of vitamin A and β-carotene have been seen in AD patients [23, 24], and a higher β-carotene plasma level was associated with better memory performance [25]. Data on the supplementation of vitamin A alone in AD were not available. 

#### 3.1.2. Vitamin C

Vitamin C has been proven to reduce Aβ oligomer formation and oxidative stress in vitro and in vivo studies [26, 27]. Data from cohort studies about the supplement effect of vitamin C on AD are conflicting. A prospective study (*n* = 980) evaluating the relationship between 4 years of vitamin C and vitamin E intakes and the incidence of AD showed no difference in the incidence of AD during the 4-year followup [28], and the same relationship was also found in another prospective study (*n* = 5395) showing that vitamin C intake was not associated with AD risk [29]. However, results from mostly published prospective observational studies (*n* = 4740) suggested that the combined use of vitamin C and vitamin E for at least 3 years was associated with the reduction of AD prevalence and incidence [30]. Overall, there is a large body of evidence that maintaining healthy vitamin C levels can have a protective function against AD, but avoiding vitamin C deficiency is likely to be more beneficial than taking supplements on top of a normal, healthy diet [31]. 

#### 3.1.3. Vitamin E

Vitamin E is a lipid-soluble antioxidant that has been found to confer neuroprotection by inhibiting oxidative stress [32–34] and scavenging Aβ-associated free radicals [35]. Compared to cognitive normal subjects, AD and mild cognitive impairment (MCI) had lower levels of total tocopherols, total tocotrienols, and total vitamin E [36]. When considered each vitamin E form alone, intake of *α*-tocopherols and *γ*-tocopherols was associated with a slower rate of cognitive decline [37]. However, in a double-blind, randomized controlled study with 769 subjects, there were no significant differences in the probability of progression to AD in the vitamin E group compared to the placebo group [38]. There were limitations to the study, for example, the forms of vitamin E were not clarified. In addition, the composition of vitamin E supplement might not reflex the actual composition in the diet. At present, there is no reliable evidence of efficacy of vitamin E in the prevention or treatment of people with AD; thus, more research is needed [39]. 

#### 3.1.4. Selenium

Current knowledge provides no evidence of a role of selenium (Se) in the treatment of AD but allows speculation on a potential preventive relevance [40], and selenium has been reported to play an important role in the antioxidative defense [41, 42]. AD patients showed a significant lower Se level in plasma, erythrocytes, and nails when compared to controls [43]. Several interventional trials demonstrated that supplementations of selenium-containing mixtures improved cognition [44–46]; however, as the authors did not specify the form of Se, the validity of the interventional trials was limited. In addition, the window of selenium's biological efficacy is a narrow one. The relationship between Se supplementation and AD requires confirmation by randomized trials.

#### 3.1.5. Polyphenols

Polyphenols are natural antioxidants that provide protective effects to AD through a variety of biological actions, such as interaction with transition metals, inactivation of free radicals, inhibition of inflammatory response, modulation in the activity of different enzymes, and effects on intracellular signaling pathways and gene expression [47–49]. Several animal studies have demonstrated that polyphenols inhibited Aβ formation and attenuated cognitive deterioration [50–54]. Data from a randomized, double-blind controlled clinical trial of polyphenols supplementation in 100 subjects showed that polyphenols contained in antioxidant beverages might benefit AD patients by decreasing homocysteine concentrations in AD patients [55]. 

### 3.2. B Vitamins (Folic Acid, Vitamin B6, and Vitamin B12)

B vitamins might contribute to AD by inhibiting oxidative stress and lowering the concentrations of homocysteine [56, 57]. Vitamin B6 has been reported to inhibit oxidative stress in AD [56]. High concentrations of homocysteine have been linked to an increased risk of AD [58–60], and homocysteine was significantly elevated in AD patients [61]; high dose supplementation of vitamin B6, B12, and folate lowers plasma homocysteine concentrations in AD patients [62]; homocysteine-lowering treatment might be a therapeutic target for AD.

In a cohort of 816 subjects, low serum folate concentrations were reported to increase the risk of AD [59], and increased dietary intake of folate decreased the risk of AD [72, 73]. A meta-analysis of 9 folic acid supplements versus placebo in 2,835 participants suggested that folic acid, with or without other B vitamins, had no effect on cognitive function within 3 years of the start of treatment [74]. In a randomized controlled trial of homocysteine-lowering treatment with B vitamins, 140 subjects with mild-to-moderate AD or vascular dementia were assigned to take 1 mg methylcobalamin and 5 mg folic acid, or placebo once daily for 24 months; there was no significant group difference in changes in any of the neuropsychological scores [75]. However, in another randomized controlled trial, 266 participants with MCI were randomly assigned to receive a daily dose of 0.8 mg folic acid, 0.5 mg vitamin B12, and 20 mg vitamin B6, or placebo for 2 years; the mean plasma homocysteine concentration was 30% lower in those treated with B vitamins relative to placebo there was significant benefit of B vitamin treatment among participants with baseline homocysteine above the median (11.3 *µ*mol/L) in global cognition, episodic memory and semantic memory [76]. The reasons for the discrepancy in findings for the effect of B vitamins AD and cognition might be the nonuniform choice of regimen, the different pathological courses of the patients, and the diverse measurements of the results. Thus, the available evidence, so far, is insufficient to draw definitive conclusion on the association of B vitamins with cognitive decline [77]. 

### 3.3. Vitamin D

 Vitamin D might have little association with Aβ mechanisms and its potential association with AD might involve other pathways, such as antioxidative, vascular, anti-inflammatory, or metabolic pathways [78]. Genetic studies have provided the opportunity to determine that vitamin D is associated with AD risk [79, 80].

 A meta-analysis of 10 studies showed that AD cases had lower serum vitamin D concentrations than matched controls [81]. In a study with 1,604 men, little evidence of associations between lower 25-hydroxyvitamin D level and cognitive function was found [82]; however, data from another large population-based study with 5,596 community-dwelling women showed that women with inadequate intakes had a lower mean Pfeiffer Short Portable Mental State Questionnaire (SPMSQ) score compared to women with recommended weekly vitamin D dietary intakes [83]. The cross-sectional association between vitamin D and cognition strengthened the hypothesis that correcting hypovitaminosis D among older adults could prevent cognitive decline but prevent the finding of a cause and effect link. Randomized controlled trials testing vitamin D supplements versus placebo should be the next step [84]. 

### 3.4. Metals 

 Dysfunctional homeostasis of transition metals is believed to play a role in the pathogenesis of AD by forming reactive species through metal amyloid complexes [85, 86]. Modulating metals has been proposed as a therapeutic strategy for AD [87]; bivalent cation chelators such as clioquinol and its later derivatives are being developed as a novel AD drug [88]. 

#### 3.4.1. Copper

Copper is essential for life, but in excess can be toxic. High dietary intake of copper in conjunction with a diet high in saturated and transfats was reported to be associated with cognitive decline [89, 90]. 

A meta-analysis of 17 studies with 1425 subjects showed that AD patients have higher levels of serum copper than controls [91]. Copper dysfunction is thought to play a role in AD pathology [92]; however, data from a prospective, randomized, placebo-controlled trial with 68 subjects showed that oral copper supplementation had neither a detrimental nor a promoting effect on the progression of AD [93]. 

#### 3.4.2. Iron

 Iron mediates the oxidative stress in AD, and an imbalance in iron homeostasis is thought as a precursor to AD [94, 95]. Diets excessive in Fe together with a high intake of saturated fat acids have been recommended to be avoided in the elderly [89]. However, iron supplementation has been reported to improve attention and concentration irrespective of baseline iron status in older children and adults [96]. 

#### 3.4.3. Zinc

 Zinc supplementation was found to reduce both Aβ and tau pathologies in the hippocampus and to delay hippocampus-dependent memory deficits in AD mouse model [97]. Zinc deficiency was reported to be associated with cognition loss in AD patients [98].

### 3.5. Fats

 Different consumption levels of the major specific fat types, rather than total fat intake itself, appeared to influence cognitive aging. Higher monounsaturated fatty acid was related to better cognitive function, while higher saturated fatty acid was associated with worse cognitive function. Total fat, polyunsaturated fatty acid, transfat intakes were not associated with cognition changes [99].

#### 3.5.1. Monounsaturated Fatty Acids

Monounsaturated fatty acids (MUFAs) and MUFA derivatives have anti-inflammatory effects in vivo [100, 101], and derivatives of MUFA, including low molecular weight phenols, were reported to have antioxidant effects [102]. Data from a prospective study suggested that higher intake of monounsaturated fatty acid is associated with less cognitive decline [103].

#### 3.5.2. Polyunsaturated Fatty Acids (Omega-3 Polyunsaturated Fatty Acids)

*C*urrent evidence suggests that elevated intake of polyunsaturated fatty acids might be beneficial to AD [104–106]. Dietary supplementation of omega-3 polyunsaturated fatty acids was reported to affect expression of genes that might influence inflammatory process [107]; however, the protective effects might be limited to APOE epslion4 noncarriers [108]. Dacosahexaenoic acid (DHA), the main form of omega-3 fatty acids, has been demonstrated to reduce Aβ production and pathological changes in AD animal models [109–112]. A meta-analysis of 11 observational studies and 4 clinical trials showed that omega-3 fatty acids slowed cognitive decline in elderly individuals without dementia [113]. However, data from randomized controlled trials showed that supplementation with DHA and eicosapentaenoic acid (EPA), compared with placebo, did not slow the rate of cognitive decline and functional decline [114–116]. The contradictory results between observational studies and randomized controlled trials might be that the duration of randomized controlled trials was often not long enough. 

#### 3.5.3. Saturated Fatty Acids

 Elevated intake of saturated fatty acids could have negative effects on cognitive functions [117]. In a study of 1,449 participants with an average followup of 21 years, moderate intake of saturated fatty acids was associated with an increased risk of AD and dementia, especially among APOE epslion4 carriers, whereas a higher intake did not affect the risk [118], suggesting that there may be a threshold association.

#### 3.5.4. Transfatty Acid

 Transfatty acid might potentially increase AD risk or cause an earlier onset of the disease by increasing the production of Aβ through increase of amyloidogenic and decrease of nonamyloidogenic processing of amyloid precursor protein [119]. However, in a prospective study with 482 women over a followup of 3 years, a validated food frequency was administrated twice to assess dietary intake before cognitive assessment; greater intake of transfat was not associated with cognitive decline [103]. No reliable data from randomized trials on the association of transfatty acids with AD were available. 

### 3.6. Carbohydrates 

It has been suggested that patients with T2DM (type 2 diabetes mellitus) are at an increased risk of getting AD [5]. Deficient brain insulin signaling pathway has been proposed as the common mechanism in the two disorders [120]. In AD patient brains, reduced insulin levels, insulin receptor expression, and insulin resistance have been reported [121–123]. With increased exposure to glucose, multiple proteins in neurons are susceptible to glycation, which is viewed as being an important contributor to AD [124]. Therefore, a diet high in carbohydrates may be detrimental to AD [125, 126]. However, in a prospective study with 939 participants over 6.3 years of followup, glycemic load reflexing carbohydrate content in food was not associated with a higher risk of AD [127]. No reliable data from randomized trials on a diet high in carbohydrate and AD were available. 

## 4. The Effects of Foods and Beverages on the Risk of AD

**Figure 1:**
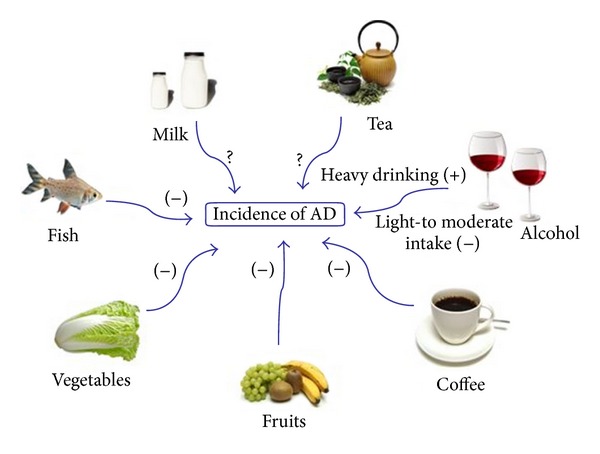
Foods and beverages that influence the incidence of AD. Fish, vegetables, fruits, coffee, and light-to-moderate alcohol intake are reported to reduce AD incidence. Milk and tea are reported to influence cognition, but their influence on AD is not clear.

Single nutrients are not consumed in isolation but as a part of diet; examining the role of single nutrients is complicated and difficult due to the interaction between nutrients. Therefore, examining foods rather than single nutrients might be more useful, and many foods and beverages have been reported to affect the risk of AD (Figure 1). 

### 4.1. Fish

 Epidemiological studies suggest that fish consumption can reduce the risk of dementia and AD, especially among APOE epslion4 non-carriers [128–132]. The positive link is thought to be associated with marine long chain omega-3 fatty acids, EPA, and DHA. The belief that consumption of fish as a whole is gaining popularity. In a prospective study with 815 participants aged from 65 to 94 years, consumption of fish more than once a week had 60% less risk of AD compared with those who rarely or never ate fish [131]. Data from randomized trials of the effect of whole-fish consumption on the risk of AD were not available.

### 4.2. Fruits and Vegetables

 Frequent consumption of fruits and vegetables might decrease the risk of AD and dementia [128]. A medium or great proportion of fruits and vegetables in the diet, compared with no or small proportion, was associated with a decreased risk of AD and dementia [133]. If the association between fruits and vegetables intake and AD is validated, the mechanism might be that fruits and vegetables are rich sources of antioxidants and bioactive compounds (e.g., vitamin E, vitamin C, carotenoids, and flavonoids) and also low in saturated fats [134].

 Higher vegetable, but not fruit, consumption was reported to be associated with slower rate of cognitive decline in a cohort of 3,718 participants aged 65 years and older; among types of vegetables, green leafy vegetables had the strongest association [134]. The paradoxical results might be due to that vegetables, especially green leafy vegetables, contain more vitamin E than fruits, and some unknown dietary component offsets the protective effects of antioxidants in fruits. In another cohort of 2,613 participants aged 43–70 years old, total intakes of fruits, legumes, and juices were not associated with change in cognitive cognition, while higher intakes of some subgroups (e.g., nuts, cabbage, and root vegetables) may diminish age-related cognitive decline in middle-aged individuals [135]. Data from randomized controlled trials were not available. 

### 4.3. Dairy

 A lower consumption of milk or dairy products was found to be associated with poor cognitive function [136, 137]. Dairy, rich in vitamin D, phosphorus, and magnesium may reduce the risk of cognitive impairment by decreasing vascular alterations and structural brain changes that occur with cognitive decline [136]. However, the consumption of whole-fat dairy products may be associated with cognitive decline in the elderly [136]. Moderate intake of unsaturated fats from milk products and spreads at midlife decreased the risk of AD, while saturated fat intake from milk products and spreads at midlife was associated with an increased risk of AD [118, 138]. Unfortunately, the observational studies examined diary as a component of dietary intake not as their primary focus, and there was no evidence available from randomized controlled trials.

### 4.4. Coffee

Coffee drinking may be associated with a decreased risk of AD [139]. A trend towards a protective effect of caffeine on AD was reported [140, 141]. Coffee may be the best source of caffeine to protect against AD due to a component in coffee that synergizes with caffeine to selectively enhance plasma cytokines [142]. A quantitative review of four studies (two case-control studies and two cohorts) showed that coffee consumption is inversely associated with the risk of AD, compared to nonconsumers; the risk estimate of AD in coffee consumers is 0.70 with 95% confidence interval 0.55–0.90 [143]. However, the four studies had heterogeneous methodologies and results, so further prospective studies evaluating the consumption of coffee and AD are strongly needed.

### 4.5. Tea

Observational studies suggest that tea drinking was associated with lower risks of cognitive impairment and decline [144, 145], and the protective effect was not limited to a particular type of tea [144]. Black tea was shown to significantly enhance auditory and visual attention compared to placebo [146]. Green tea polyphenols may inhibit cognitive impairment via modulating oxidative stress [147–149], and green tea epigallocatechin-3-gallate (EGCG) has been shown to reduce β-amyloid generation and sarkosyl-soluble phosphorylated tau isoforms in AD mouse models [150, 151]. The neuroprotective effects of tea consumption could be due to catechins, L-theanine, polyphenols, and other compounds in tea leaves [152]. Therefore, tea might be a relevant contributor to AD. 

### 4.6. Alcohol

Epidemiological studies suggest that light-to-moderate alcohol intake was associated with a reduced risk of AD, particularly among APOE epslion4 non-carriers [153–155]. However, heavy drinking (>2 drinks), alongside with heavy smoking and APOE epsilon4, was associated with an earlier onset of AD [156]. The mechanisms by which low-to moderate intake could be protective against AD while heavy intake was detrimental to were unclear.

Different types of alcohol (wine, beer, and mixed alcohol beverages) may have different effects on AD. Resveratrol and other polyphenols in red wine have been found to diminish plaque formation and protect against Aβ-induced neurotoxicity [157–159], and moderate beer consumption was thought to afford a protective factor for AD due to its content in bioavailability silicon [160]. Therefore, alcohol intake might provide benefits to AD, but the quantity and type of alcohol were not clear.

## 5. The Effects of Dietary Patterns on the Risk of AD (Figure 2)

 Dietary pattern, a combination of food components that summarizes an overall diet for a study population, can have various effects on cognitive function and AD (Table 1). A dietary pattern, characterized by a high intake of meat, butter, high-fat dairy products, eggs, and refined sugar, has been found in AD patients [161]. 

### 5.1. Western Diet

A Western diet is characterized by higher intake of red and processed meats, refined grains, sweets, and desserts [162]. A high-fat Western diet may contribute to the development of AD by impacting Aβ deposition and oxidative stress [163, 164]. Data from epidemiological studies exploring Western diet and the risk of AD were not available. 

### 5.2. Japanese Diet

Traditional Japanese diet is characterized by increased intake of fish and plant foods (soybean products, seaweeds, vegetables, and fruits) and decreased intake of refined carbohydrates and animal fats (meat) [165]. In a population-based study with a total of 1006 Japanese subjects followed by 15 years, a dietary characterized by a high intake of soybeans and soybean products, vegetables, algae, and milk and dairy products and a low intake of rice was associated with a reduced risk of AD [63]. 

### 5.3. Health Diets 

In a cohort of 3054 participants, a healthy diet was defined as one positively correlated with consumption of fruit, whole grains, fresh dairy products, vegetables, breakfast cereal, tea, vegetable fat, nuts, and fish and negatively correlated with meat, poultry, refined grains, animal fat, and processed meat. Participants with the highest compared with lowest adherence to the health diet had a better cognitive function [64]. A healthy diet, characterized by higher consumption of fish by men and fruits and vegetables by women, was also reported to be associated with better cognitive performance [65]. In another study with 525 subjects, a healthy diet index was constructed to assess healthy and unhealthy diet components; persons with a healthy diet (healthy-diet index >8 points) had a decreased risk of AD [66]. 

### 5.4. DASH-Style Diets 

The Dietary Approaches to Stop Hypertension (DASH) diet contains a high intake of plant foods, fruits, vegetables, fish, poultry, whole grains, low-fat dairy products, and nuts, while minimizing intake of red meat, sodium, sweets, and sugar-sweetened beverages [165]. In a randomized clinical trial of 124 participants with elevated blood pressure, subjects on the DASH diet exhibited greater neurocognitive improvements when compared to normal subjects [67]. As hypertension is associated with increased risk for AD [166], it is biologically plausible that DASH could reduce the risk of AD. 

### 5.5. Mediterranean Diets 

The Mediterranean diet, a typical diet of the Mediterranean region, is characterized by a high consumption of fruits, vegetables, cereals, bread, potatoes, poultry, beans, nuts, olive oil, and fish; a moderate consumption of alcohol; a lower consumption of red meat and dairy products.

 Adherence to the Mediterranean diet may not only affect the risk of AD but also mortality in AD [167]. A meta-analysis of eighteen cohort studies with 2,190,627 subjects showed that adherence to the Mediterranean diet was associated with a significant reduction of overall mortality and neurodegenerative diseases [168]. Several researches supported a beneficial association between adherence to a Mediterranean diet and AD [68, 69]. As fruits, and vegetables, fish, and moderate alcohol reduced the risk of AD [128, 130, 131, 153–155], in spite of lack of data from randomized controlled trials, the Mediterranean diet can be thought to be beneficial to AD.

## 6. Conclusions and Future Directions 

In this paper, we searched PubMed articles published from 2000 to 2013, using the search terms “Alzheimer's disease,” “nutrition,” “nutrients,” “food,” “diet,” “dietary patterns,” “overweight,” “obesity,” “prospective cohort studies,” “randomized controlled trials,” “systematic review,” and “meta-analysis.” Articles were also identified through searches of lists. Studies were selected for inclusion on the basis of a judgment about the quality of the evidence according to four key elements: study design, study quality, consistency, and directness, as proposed by the Grading of Recommendations Assessment, Development and Evaluating (GRADE) working group. For each nutrient, food, or dietary pattern, only the studies with the highest level of evidence were included. If randomized trials had not been undertaken and only observational data were available, studies were included if they were prospective, population-based, and large, with standardized diagnostic criteria for AD. Studies were excluded if serious limitations to study quality and major uncertainty about directness existed. Only articles published in English were included. 

Epidemiological studies suggest antioxidants, vitamins, polyphenols, polyunsaturated fatty acids, fish, fruits, vegetables, tea, and light-to moderate consumption of alcohol are beneficial for AD, while trans-fatty acids, saturated fatty acids, carbohydrates, and whole-fat dairy are detrimental to AD. However, epidemiological studies cannot eliminate bias and confounding in any association between a risk factor and AD [169]; the results of such studies should be interpreted with caution. In addition, it is difficult to examine the individual effects of nutrients and foods because they are correlated with each other; therefore, the idea of focusing on diet as a whole is gaining momentum. 

 Randomization is the best method to minimize bias and confounding and establish causality. However, randomized trials are not always feasible, and the few randomized controlled trials that have been undertaken provide conclusions that dietary supplementation of vitamin E, B vitamins, and polyunsaturated fatty acids does not reduce cognitive decline and the risk of AD. Several reasons might be responsible for the discrepancy between observational studies and randomized controlled trials. First, nutrients might be useful only for primary prevention of AD, and not protective once the pathological process started. Second, the dose of nutrients might not be equivalent to levels seen in the epidemiological studies. Third, the duration of most trials has been suggested to be inadequate to show benefits. Besides, the association between nutrients and epidemiological studies is confounded by social and behavioural factors acting across the life course [170]. 

 Dietary pattern, which better reflexes the complexity of diet, has emerged in recent years to examine the relationship between diet and AD. Adherence to the Mediterranean diet, the Japanese diet, and the healthy diet has been reported to be associated with decreased risk of AD. Given that the studies relating dietary patterns to AD are very few, further studies are needed. In general, the very few studies that have been done suggested that higher intake of fruits, vegetables, fish, nuts, legumes, cereal, lower intake of meats, high fat diary, sodium, sweets, and refined grains seemed to be associated with reduced risk of AD.

 Further researches are needed to improve the quality of evidence relating to the association of many nutrients, foods, and dietary patterns with AD. To establish a causative role for specific nutrients, foods, and dietary patterns in the pathogenesis of AD, adequately powered, large randomized trials are needed in which the patient population and intervention are carefully described. 

## Figures and Tables

**Figure 2:**
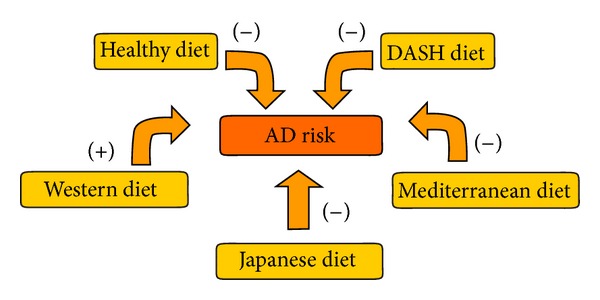
Dietary patterns that influence the risk of AD. Healthy diet, DASH-diet, Mediterranean diet, and Japanese diet might decrease the risk of AD. Western diet might increase the risk of AD. DASH diet: the Dietary Approaches to Stop Hypertension.

**Table 1:** Summary of studies linking dietary pattern to cognitive function and AD.

Reference	Participants	Design	Result
Ozawa et al. [63]	1006 Japanese community	Cohort	A higher adherence to a dietary pattern characterized by a high intake of soybeans and soybean products, vegetables, algae, and milk and dairy products and a low intake of rice is associated with dementia in the general Japanese population.

Kesse-Guyot et al. [64]	3054 participants	Cohort	The healthy pattern was associated with better global cognitive function (50.1 ± 0.7 versus 48.9 ± 0.7; *P* trend = 0.001) and verbal memory (49.7 ± 0.4 versus 48.7 ± 0.4; *P* trend = 0.01).

Samieri et al. [65]	1724 elderly community dwellers	Cohort	A “healthy” cluster characterized by higher consumption of fish by men and fruits and vegetables by women had a significantly lower mean number of errors to Mini Mental State score after adjustment for sociodemographic variables (beta = −0.11; 95% confidence interval (CI), −0.22 to −0.004 in men; beta = −0.13; 95% CI, −0.22 to −0.04 in women).

Eskelinen et al. [66]	525 subjects	Cohort	Persons with a healthy diet (healthy-diet index >8 points) had a decreased risk of AD (OR 0.08, 95% CI 0.01–0.09) compared to persons with an unhealthy diet (0–8 points)

Smith et al. [67]	124 participants with elevated blood pressure	Randomized controlled trial	DASH diet combining aerobic exercise and caloric restriction improves neurocognitive function among sedentary and overweight/obese individuals with prehypertension and hypertension.

Scarmeas et al. [68]	2258 nondemented individuals	Case control	Higher adherence to the MeDi was associated with lower risk for AD (odds ratio, 0.76; 95% confidence interval, 0.67–0.87; *P* < 0.001). Compared with subjects in the lowest MeDi tertile, subjects in the middle MeDi tertile had an odds ratio of 0.47 (95% confidence interval, 0.29–0.76) and those at the highest tertile had an odds ratio of 0.32 (95% confidence interval, 0.17–0.59) for AD (*P* for trend <0.001).

Scarmeas et al. [69]	1393 cognitively normal participants	Cohort	Higher adherence to the MeDi is associated with a trend for reduced risk of developing MCI and with reduced risk of MCI conversion to AD.

Féart et al. [70]	1410 adults aged over 65 y	Cohort	Higher adherence to a Mediterranean diet was associated with slower MMSE cognitive decline but not consistently with other cognitive tests. Higher adherence was not associated with risk for incident dementia.

Tangney et al. [71]	3790 participants aged over 65	Cohort	The Mediterranean dietary pattern may reduce the rate of cognitive decline with older age.
